# Not Your Typical Mucocele: A Case Report of a Benign Sigmoidal Diverticular Mucocele

**DOI:** 10.7759/cureus.7743

**Published:** 2020-04-20

**Authors:** Daniel Alcantar, Fanny Giron, Layth Al-Jaashaami, Rashmi Kumar

**Affiliations:** 1 Internal Medicine, MacNeal Hospital, Berwyn, USA; 2 Gastroenterology, Banner University Medical Center, Phoenix, USA

**Keywords:** mucocele, diverticulum, colonoscopy

## Abstract

In recent literature, mucoceles have been discovered to be in the appendix vermiformis or in the nasal sinuses. Although rare, colonic mucoceles, as well as rectal mucoceles, have also been encountered. Furthermore, colonic mucoceles arising from a diverticulum is an even more unusual occurrence, and to date, there has been only one reported case. We present a 48-year-old male with a past medical history of multiple episodes of diverticulitis who presented to the emergency department complaining of bilateral lower quadrant abdominal pain for three days. Upon arrival to the emergency department, the patient had a CT scan of the abdomen and pelvis, which showed an annular constricting 65 mm mass in the proximal sigmoid causing large bowel obstruction. The patient underwent unsuccessful endoscopies and inevitably underwent a hand-assisted laparoscopic sigmoid resection. The following days, the biopsy returned and resulted to be a mucocele arising from a sigmoid diverticulum. We encountered the very first benign colonic mucocele arising from a sigmoid diverticulum.

## Introduction

In recent literature, mucoceles have been discovered to be in the appendix vermiformis or the nasal sinuses [1]. Although rare, colonic mucoceles, as well as rectal mucoceles, have also been encountered [2,3]. Furthermore, colonic mucoceles arising from a diverticulum is an even more unusual occurrence, and to date, there has been only one reported case [4]. We present a rare case of a benign obstructing mucocele arising from a sigmoid diverticulum, likely secondary to multiple bouts of diverticulitis.

## Case presentation

**Figure 1:**
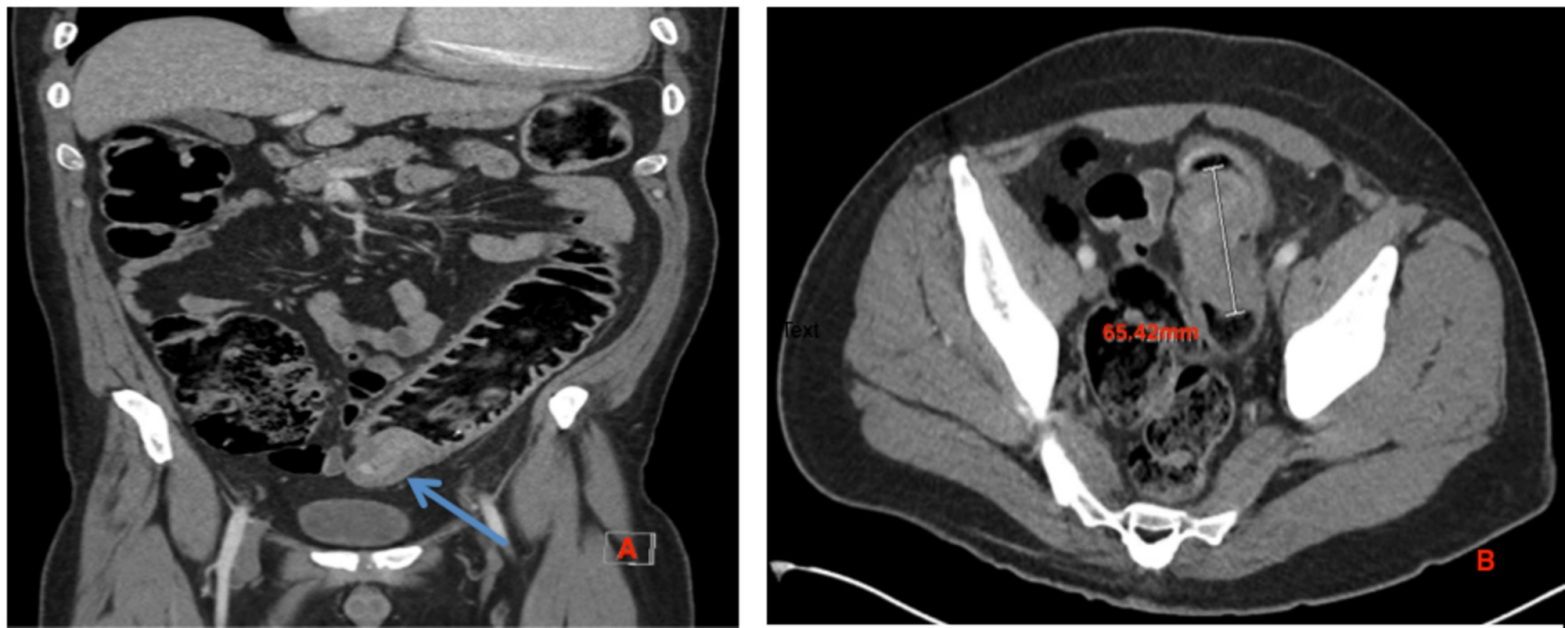
(A) A coronal view of CT of the abdomen and pelvis showing large obstructing mucocele (blue arrow). (B) An axial view of CT of the abdomen and pelvis showing a 65-mm mucocele.

A 48-year-old male with a past medical history of multiple episodes of diverticulitis presented to the emergency department complaining of bilateral lower quadrant abdominal pain for three days. Upon arrival to the emergency department, the patient had an extensive workup, including a CT scan of the abdomen and pelvis, which showed an annular constricting 65 mm mass in the proximal sigmoid causing large bowel obstruction (Figure 1). Because of this, the gastroenterology (GI) service was consulted.

Three months before the current admission, the patient presented with similar complaints, for which he was found to have a similar-appearing mass on the CT scan. At that time, he underwent an unsuccessful flexible sigmoidoscopy (flex-sig) as unable to pass the large sigmoidal mass. Biopsies were taken at that time and resulted to be an inflammatory polyp with surface erosion, and hyperplastic mucosal changes with no dysplasia or malignancy noted. The patient was discharged home and was asked to follow up with gastroenterology as well as colorectal surgery as an outpatient. Unfortunately, the patient was lost to follow-up.

**Figure 2:**
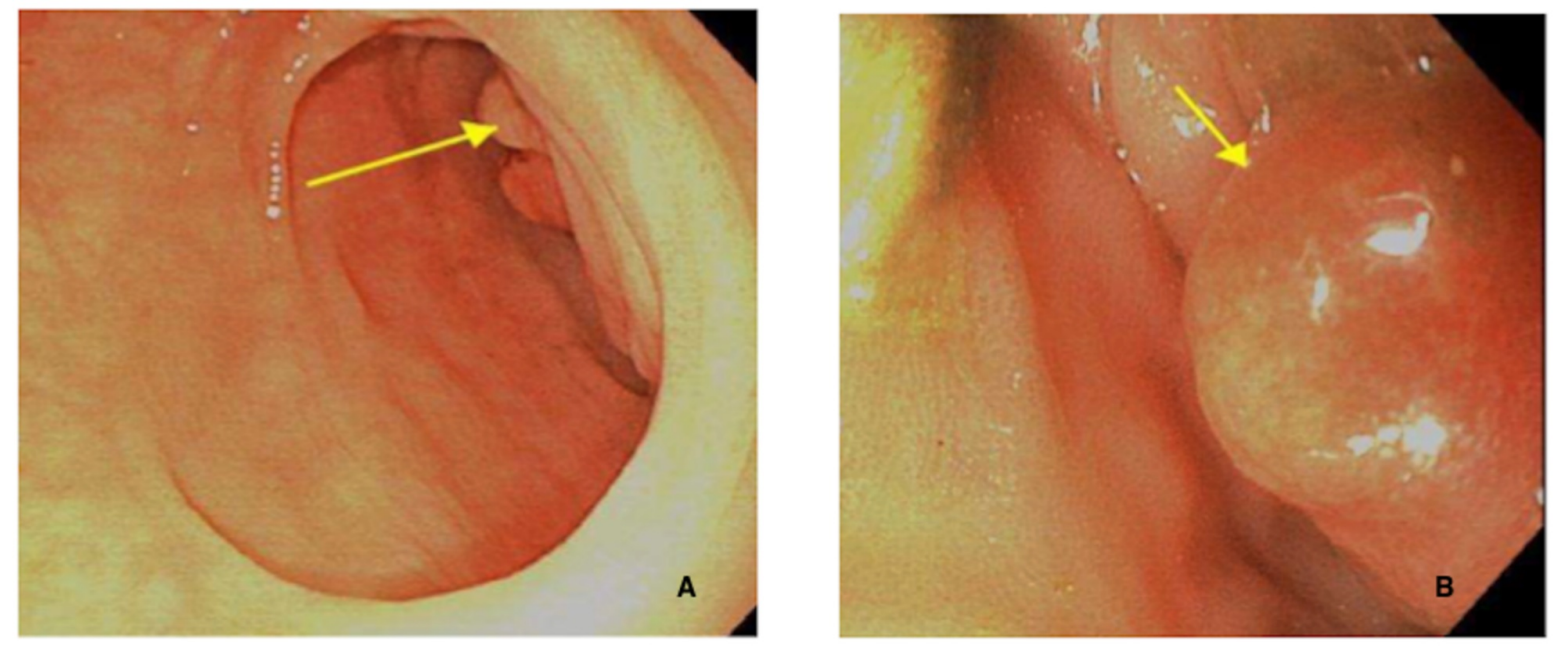
(A) Large mucocele causing obstruction (yellow arrow). (B) Colonscopy attempting to bypass mucocele (yellow arrow).

Because of the patient’s history, the GI service decided to attempt another flex-sig with the possible use of a pediatric gastroscope (in case of an unsuccessful pass with flex-sig). Unfortunately, despite adequate bowel prep, unsuccessful passes with the flex-sig and pediatric gastroscope were performed (Figure 2). Biopsies again were taken; however, only proximal portions of the mass were biopsied. The biopsies collected reported colonic mucosa with an increase in lamina propria, inflammatory infiltrate, and mild architectural distortion. Because of two unsuccessful endoscopies and inadequate biopsies, the colorectal surgery service was consulted and recommended surgery.

**Figure 3:**
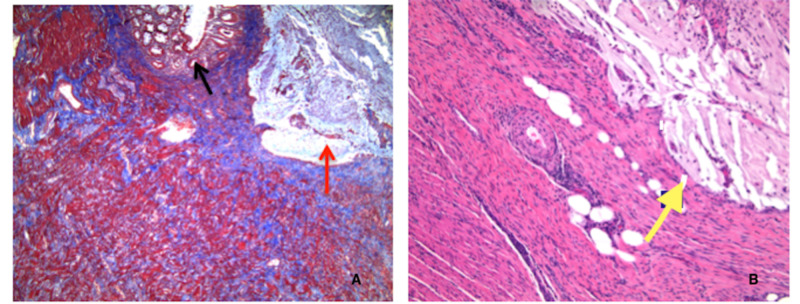
(A) Trichrome stain showing the diverticulum (black arrow) and mucocele (red arrow). (B) High-power image of smooth muscle wall with mucin (yellow arrow).

Within the following day, the patient underwent hand-assisted laparoscopic sigmoid resection. Postoperatively, the patient was hemodynamically stable and was passing flatus with bowel movements and the patient was discharged home. The pathology report confirmed a large mucocele (Figure 3) of the sigmoid colon with mucosal prolapse and hypertrophic prolapse. Within the specimen included 14 benign pericolic lymph nodes.

## Discussion

A mucocele is a rare pathologic entity that can be associated with the gallbladder, appendix, and craniofacial sinuses [1]. Appendiceal mucoceles occur only in 0.1%-0.3% of all appendectomies and more than 70% of these are discovered incidentally [5]. Conversely, colonic mucoceles are extremely rare manifestations, with very few cases being reported [2,4,5]. Interestingly, most cases of colonic mucoceles were encountered post bowel resection and the unused bowel segment was a possible predilection site of mucin accumulation [2].

The diagnosis of a mucocele can be difficult because of its rarity and non-specific imaging findings. There is no method to determine the diagnosis with certainty and uncommonly, they may coexist with other rare lesions, such as intestinal endometriosis [5]. In regards to treatment, studies have recommended surgical removal if the lesion is greater than 2 cm, whereas there has been one case where a lesion less than 2 cm was removed endoscopically via polypectomy [6,7]. In our case, the large sigmoidal mass was discovered incidentally via the CT scan. Due to its size and large bowel obstructive symptoms, it was resected.

The etiology of a mucocele is still in question; however, several mechanisms have been described. Studies have hypothesized that appendiceal mucoceles occur due to cystic dilatation of the appendiceal lumen and accumulation of mucin within the lumen [5]. In regards to colonic mucoceles, it has been suggested that atrophic changes develop, resulting in narrowing of the luminal diameter. Because of this narrowing, viscous mucin then passes through the stricture where the mucocele developed [2]. We believe, given his history of multiple bouts of diverticulitis, one of the diverticula may have become sequestered, leading to a gross mucinous accumulation forming a mucocele.

The overall prevalence of diverticulosis increases with age. Geographically, the location of the diverticula differs as well. In Western countries, most diverticular disease occurs in the sigmoid colon. In contrast, in Asia, right-sided diverticular disease is predominant. The risk of being hospitalized for diverticulitis is three times higher than that associated with diverticular bleeding. Diverticulitis is more common in patients aged 18 to 80 years than is diverticular bleeding, and is more prevalent in women than in men [8]. The overall prevalence of hospitalization increased from 74.1 per 100,000 persons in 2010 to 91.9 per 100,000 in 2010 [9]. This increase was noted in the age group of 17 to 70 years [9].

Because of our patient’s history of multiple flares of diverticulitis, it can be hypothesized that the mucocele may have surfaced from a sigmoid diverticulum due to the multiple flares of diverticulitis in the past. If this holds true, this would be considered the second mucocele encountered in a colonic diverticulum, the first in being the United States. The first case reported was in Japan by Nakatani et al., who describes a low-grade mucinous neoplasm that was present in a cecal diverticulum [4]. When comparing our case with the Nakatani’s case, our case was encountered on the left side of the colon in contrast to the right. Furthermore, our patient had a history of multiple flares of diverticulitis, which is not reported in the cecal case. Lastly, our mucocele case was benign.

## Conclusions

We encountered the very first benign colonic mucocele arising from a sigmoid diverticulum. Although there is no clear diagnostic or treatment criteria in place, as of yet, it appears complete resection is favorable in order to obtain full histological examination. Further studies are warranted in order to build evidence on proper diagnosis and treatment.
